# Effect of dietary patterns on oxidative stress in Patiants with metabolic syndrome: Tehran Lipid and Glucose Study

**DOI:** 10.22088/cjim.9.4.376

**Published:** 2018

**Authors:** Parvin Mirmiran, Hoda Hadavi, Azadeh Mottaghi, Fereidoun Azizi

**Affiliations:** 1 Nutrition and Endocrine Research Center, Research Institute of Endocrine Sciences, Shahid Beheshti University of Medical Sciences, Tehran, Iran; 2 Research Center for Prevention of Cardiovascular Diseases, Institute of Endocrinology and Metabolism, Iran University of Medical Sciences, Tehran, Iran; 3 Endocrine Research Center, Research Institute of Endocrine Sciences, Shahid Beheshti University of Medical Sciences, Tehran, Iran

**Keywords:** Dietary patterns, Oxidative stress, Metabolic syndrome

## Abstract

**Background:**

Metabolic syndrome is a prevalent condition with dramatic rising trend worldwide. Single dietary factors, such as omega-3 fatty acids consumption protect body against oxidative damage by reinforcement of dietary total antioxidant capacity but the combination of all dietary components may be more effective when studied as integrated dietary patterns. This present study was designed to assess the association between different dietary patterns and oxidative stress in a population of Tehranian adults suffering from metabolic syndrome.

**Methods:**

Dietary data were collected using a validated 147-item semi-quantitative FFQ with a standard serving size. Factor analysis method was used to derive dietary patterns. Blood analysis and anthropometric measurements were also obtained. Oxidative stress was assesses using serum levels of Malondialdehyde (MDA) and total antioxidant capacity (TAC).

**Results:**

The regression coefficient for TAC and MDA with different quintiles of dietary patterns, adjusted for potential confounder in model 3 reveal a significant positive association between healthy pattern and serum TAC levels (β=0.244, p=0.008) and also between serum MDA levels and the unhealthy pattern (β=0.387, p=0.0001). On the other hand, a significant negative association found between serum TAC levels (β=-0.289, p=0.001) and the unhealthy pattern, a relationship also noted between serum MDA levels and the healthy dietary pattern (β=-0.273, p=0.002).

**Conclusions:**

Our findings suggest that following a healthy pattern filled with fruits and vegetables ameliorates oxidative stress status and on the contrary, attachment to an unhealthy pattern, characterized by higher intakes of fast foods and processed foods, aggravated the oxidative stress levels in Tehranian individuals suffering from metabolic syndrome.

Metabolic syndrome (MetS) is a prevalent condition worldwide. Its prevalence of the MetS in Europe, Asia, Australia, and North and South America ranges between 13.4% and 70.0%, by the World Health Organization (WHO) definition (1). In Iran, the age-standardized prevalence of the metabolic syndrome is about 34.7% based on the Adult Treatment Panel III (ATP III) criteria, 37.4% based on the International Diabetes Federation (IDF) definition, and 41.6% based on the ATP III/ American Heart Association (AHA)/ National Heart, Lung, and Blood Institute  (NHLBI) criteria (2). Regardless of the documented figures, it undoubtedly has dramatic rising trend, and there is the urgent need for appropriate measure to prevent and control its escalation.

Although a number of expert groups such as WHO, ATP III and European Group for the Study of Insulin Resistance (EGIR) have proposed different definitions for MetS diagnosis, their focus has been on similar criteria such as elevated fasting blood sugar (FBS) and triglyceride, high blood pressure, decreased high density lipoprotein (HDL) cholesterol and emerging obesity (3), all conditions that if not addressed early and appropriately can lead to more hazardous conditions such as heart disease, type 2 diabetes (T2D) and all cause death. Data shows that lifestyle interventions, including dietary changes and physical activity, play a crucial role in the prevention of MetS (1). Moreover, the National Cholesterol Education Program (NCEP) has already suggested dietary interventions to prevent this epidemic. 

One of the potential risk factors for some comorbidity such as MetS and T2D is oxidative stress. So, many studies are trying to find a way to detect, treat and prevent MetS with a focus on reducing oxidative stress as one of the important factors (4). It has also been proposed that MetS be ameliorated by controlling the extent of oxidative stress in the body, as oxidative stress is a phenomenon associated with pathogenetic mechanisms of numerous diseases acting by damaging important biomolecules and organs with potential impact on the whole organism (5). Results available support the concept that increased oxidative stress plays an important role in metabolic syndrome-related manifestations, including atherosclerosis, hypertension and T2D (6). 

Diets rich in whole grain cereals, fruits, and vegetables, with low animal-fat consumption, seem to confer prevention against cardiovascular disease risk factors, like hypertension, hypercholesterolemia, and obesity (7). However, most studies have examined the effects of single dietary factors, such as the hypotriglyceridemic effect of n-3 fatty acids consumption (8), protection against oxidative damage by dietary total antioxidant capacity (TAC) (9) and impact of calorie restriction (10).

Since it has been hypothesized that the combination of all dietary components may be more effective when studied as integrated dietary patterns, and also there is a lack of data on role of different dietary patterns in oxidative stress in patients with MetS, this present study was designed to assess the association between different dietary patterns and oxidative stress in a population of Tehranian adults suffering from MetS.

## Methods

**Figure 1:**
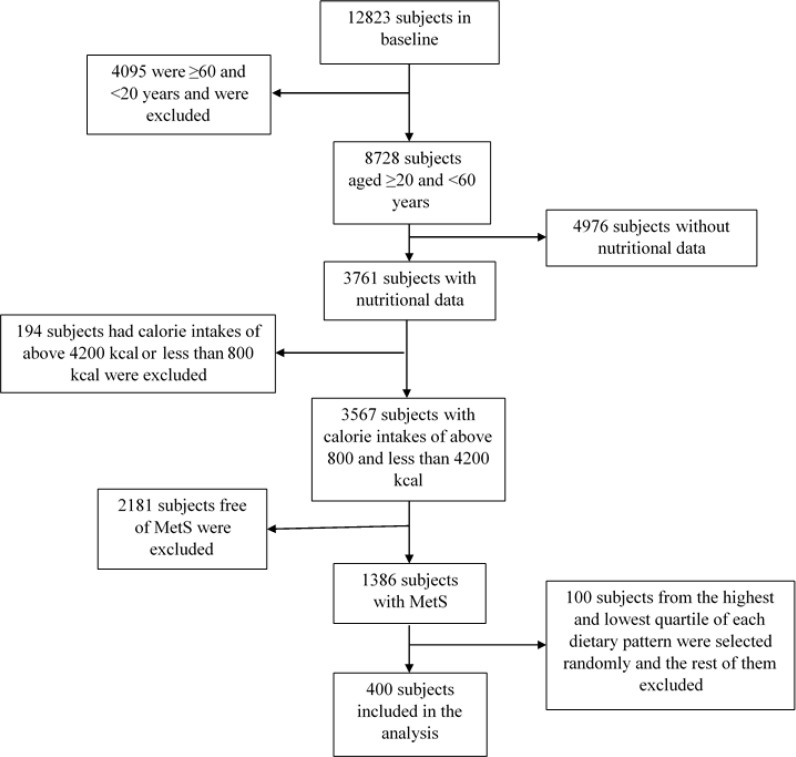
Subject sampling chart

This study was carried out within the framework of the Tehran Lipid and Glucose Study (TLGS), a prospective survey conducted on a large sample of Tehran residents. The aim of TLGS was to determine the prevalence of non-communicable disease risk factors and to encourage healthier lifestyles to ameliorate these risk factors, using data from the 4^th^ phase of TLGS. The study was approved by of the Ethics Committee of the Research Institute for Endocrine Sciences, Shahid Beheshti University of Medical Sciences and all participants provided written informed consent prior to enrollment of this study. Among 12823 people aged between 20 and 60 years in phase 4 of TLGS, the subjects diagnosed with MetS, according to ATP III criteria, were selected; those whose caloric intakes exceeded 4200 or were less than 800 kcal were excluded. Figure 1 shows subject sampling chart.

The TLGS study protocol and laboratory procedures have been published earlier (11). All subjects were interviewed individually and privately by trained personnel, using pre-tested questionnaires. Weight was measured while subjects were minimally clothed without shoes, using digital scales and recorded to the nearest 100 g; height was measured using a tape meter in a standing position without shoes, and with shoulders being in normal alignment. Body mass index was calculated as weight in kilograms divided by height in meters squared. Waist circumference (WC) was measured at the level of the umbilicus; using an un-stretched tape meter, without any pressure to the body, and was recorded to the nearest 0.1 cm. All measurements were obtained by the same person to minimize error. Krista’s physical activity questionnaire was used for assessment of physical activity levels (12). 

Metabolic equivalent (MET) was calculated according to the compendium of physical activity (13), and MET/hour was then used to energy requirement estimation. Subjects were first asked to rest for 15 minutes before their blood pressure was taken twice in a seated position by a qualified physician after one initial measurement for determining the peak inflation level using a standard mercury sphygmomanometer. There was at least 30 seconds interval between these two separate measurements; the mean of the two measurements was recorded as the participant's blood pressure. A blood sample was taken after 12–14 h of overnight fasting. All blood analyses were performed at the TLGS research laboratory on the day of blood collection. Blood samples were analyzed using a selectra 2 auto-analyzer (Vital Scientific, Spankeren, and The Netherlands). On the same day of blood collection, fasting blood sugar was also determined by the enzymatic colorimetric method using glucose oxidase. For lipid measurements, total cholesterol and triglyceride (TG) kits (Pars Azmoon, Tehran, Iran) were used. Total cholesterol and TG levels were assessed using enzymatic colorimetric tests with cholesterol esterase and cholesterol oxidase, and glycerol phosphate oxidase, respectively. HDL-C was specified after precipitation of apolipoprotein B containing lipoproteins with phosphotungstic acid; lipid standard (C.f.a.s., Boehringer Mannheim, Germany; cat. no. 759350) was used to calibrate the selectra 2 auto-analyzer for each day of laboratory analyses. 

All samples were analyzed under the internal quality control supervision of the acceptable criteria. Inter- and intra-assay coefficients of variation were 2 and 0.5% for total cholesterol and 1.6 and 0.6% for TGs, respectively. To measure Malondialdehyde (MDA) concentration, lipid peroxidation qssay kit (Abcam, Cambridge, CA, USA) was used. Total antioxidant capacity of plasma was measured using TAC assay kit (Abcam, Cambridge, CA, USA) according to manufacture protocol.

Dietary data were collected by trained dietitian with at least 5 years of experience in food consumption survey, using a validated 147-item semi-quantitative food frequency questionnaire (FFQ) (14) with a standard serving size. We analyzed nutrients using Nutritionist 4 software. We used the factor analysis method to derive dietary patterns from dietary information collected from the 22 food groups (table 1), based on similarity in nutrient contents (15). Considering data reduction in factor analysis, 2 factors were derived. The derived factors (dietary patterns) were labeled on the basis of the authors’ interpretation of data and on prior literature available (16). 

**Table 1:** Food grouping used in the dietary pattern analysis

**Food Group**	**Food items**
Refined grains	White bread (lavash, baguettes), noodles, pasta, rice, toasted bread, sweet bread, white flour, biscuits
Fast foods	Processed meats, pizza
Potatoes	Potato (all preparations)
Salty snacks	Salty biscuits, crackers, puffs, potato chips, pickles and salty vegetables, popcorn, salt
Mayonnaise	Mayonnaise, and all fatty sauces
Carbonated drinks	Coca-cola, other carbonated beverage, low-energy carbonated beverages and sweet drink, beer
Egg	Egg (all preparations)
Vegetables	All green leafy, cruciferous, yellow, tomato, and other vegetables
Whole grains	All whole and dark breads, barley, wheat, wheat germ, shredded wheat/barely, corn, biscuits prepared with whole grains
Fruit and dried fruit	All fruit and natural fruit juices, dried fruit
Poultry and fish	All fishes, canned tuna fish, chicken (all preparations)
High- and low-fat dairy products	High-fat milk and yogurt, chocolate milk, creamy yogurt, creamy cheese, ice cream, low-fat milk and yogurt, cheese
Canned products	All fruit with added sugars (Jams and fruit), honey
Liquid oils	All vegetable oils, olive oil
Solid oils	Hydrogenated fats, animal fats (butter, cream)
Sweets	All cakes, confections, chocolates, cookies, all biscuits, desserts
Red meats	Beef, lamb, hamburger
Organ meats	Liver, brain, and the other organ meats
Tea and coffee	Tea and coffee
Nuts and seeds	All nuts and seeds (raw or roasted)
Legumes	All kinds of beans, peas, lentils, soy
Olives	Olives, Olive oil

The healthy dietary pattern was high in fruits and dried fruits, olives, high- and low-fat dairy products, poultry and fish, liquid oils, and canned products, whereas unhealthy dietary pattern was dominated by carbonated drinks, fast foods, salty snacks, mayonnaise and organ meats. Dietary pattern scores were categorized into quintiles. Table 2 shows factor loading of 2 dietary patterns extracted by factor analysis. Among the participants in the 1^st^ and 5^th^ quintiles of each dietary pattern, 100 subjects who had greatest and lowest adherence to the related dietary pattern were randomly chosen, giving us a study sample of 400 subjects with MetS.

**Definition:** Patients were assessed for having MetS according to ATP III criteria (17), which diagnose MetS by the existence of three or more of the following criteria: High WC (>102 cm in men and >88 cm in women), FPG≥100 mg/dL, low levels of HDL-C (<40 mg/dL in men and <50 mg/dL in women), high TG≥150 mg/dL and high blood pressure [systolic blood pressure (SBP) ≥130 mmHg and /or diastolic blood pressure (DBP) ≥85 mmHg].

**Table 2:** Factor loading of 3 dietary patterns extracted by factor analysis

**Food Groups**	**Healthy**	**Unhealthy**
Fruit and dried fruit	0.670^a^	
Vegetables	0.721	
Olives	0.651	
Dairy products	0.392	
Liquid oils	0.273	
Solid oils	-0.556	
Canned products		0.603
Fast foods		0.702
Sweets		0.646
Mayonnaise		0.603
Organ meats		0.500
potatoes		0.283
Egg		0.386
Red meats		0.465
Refined grains	-0.477	0.233
Whole grains	-0.473	-0.468
Poultry and fish	0.405	0.200
Carbonated drinks	-0.358	0.626
Nuts and seeds	0.475	0.203
Percentage of variance explained^b^	14	15

a Values are factor loading of dietary patterns (n=1386). Factor loading ≤0.2 is not shown.

b Eigen value>1. Factor analysis was done.

**Statistical analysis:** Significant differences in baseline characteristics across quintiles of dietary pattern scores were analyzed by one-way ANOVA test. Independent sample t-test was used for assessing of any difference between quintile 1 with quintile 5 in each dietary pattern in case of MDA and TAC levels. To extract the dietary patterns, factor analysis was done. 

To determine the association of dietary patterns with biomarkers of oxidative stress, we used multiple linear regressions analysis in 3 models. Model 1 was performed with no adjustment; model 2 was adjusted for age, sex, physical activity and smoking, and finally model 3, in which all factors in model 2, plus body mass index (BMI) were adjusted. All statistical analysis was done by SPSS software (SPSS Inc., Chicago, IL, 1996, Version 20). P-values < 0.05 were considered as significant.

## Results

Table 3 shows the subjects characteristics according to quintiles of dietary pattern, indicating that younger people tend to follow the unhealthy pattern (mean age 45.11, P=001) and adherence decreases with age. Regarding sex, women had higher scores in the healthy pattern, whereas men mostly fell into the lowest quintile of this pattern (p=0001); this association for the unhealthy pattern was also significant. Adherence to unhealthy pattern increases, so do numbers of smokers (p=0.0001), indicated by inverse association between the healthy pattern and smoking (p<0.005). No significant differences between calorie intakes or either of the two patterns were observed. Participants in the highest quintile of the healthy pattern, when compared to subjects in the lowest quintile, had higher intakes of cholesterol, fiber and protein but had lower intakes of polyunsaturated fatty acids (p<0.05). 

On the other hand, subjects who had greater adherence to unhealthy pattern, compared to those with the lowest adherence, had significantly higher intakes of cholesterol, saturated fatty acids, monounsaturated fatty acids and polyunsaturated fatty acids, but had lower intakes of carbohydrates and fiber (p<0.05). Serum levels of TAC and MDA in the first and the fifth quintiles of both healthy and unhealthy patterns are demonstrated in table 4. 

Serum levels of TAC increase with greater adherence to the healthy pattern (p=0001) and there is an inverse association for followers of unhealthy patterns (p=001). There was also a significant trend for MDA and both dietary patterns, that in followers of the healthy pattern, its levels were lower, whereas elevated levels of MDA were seen in individuals with unhealthy dietary pattern (p=0.005 and 0.013, respectively). The regression coefficient for TAC and MDA with different quintiles of dietary patterns, adjusted for potential confounder in model 3 (table 5) reveal a significant positive association between healthy pattern and serum TAC levels (β=0.244, p=0.008) and also between serum MDA levels and the unhealthy pattern (β=0.387, p=0.0001). On the other hand a significant negative association found between serum TAC levels (β=-0.289, p=0.001) and the unhealthy pattern, a relationship also noted between serum MDA levels and the healthy dietary pattern (β=-0.273, p=0.002). 

**Table 3:** Basic characteristics of patients according to dietary patterns quintile

**Patients characteristics**	**Quintile of healthy pattern score**				**Quintile of unhealthy pattern score**			
	**Q1 (lowest)**	**Q3**	**Q5 (highest)**	**P value**	**Q1 (lowest)**	**Q3**	**Q5 (highest)**	**P value**
Age (y)	47.24±10.22	52.58±9.06	49.99±9.45	0.003**	53.38±9.32	49.21±9.32	45.11±7.88	0.0001***
Sex (%)				0.0001***				
Male Female	70 30	54 46	34 66		41 59	46 54	66 34	0.006**
BMI (Kg/m^2^)	30.69±4.87	29.71±2.80	31.31±4.43	0.054	30.51±3.74	31.14±5.53	30.56±4.21	0.797
Physical activity (MET-h/wk)^1^	336.08±550.97	463.30±703.08	844.23±1075.91	0.0001***	627.54±837.68	410.43±606.20	527.14±749.70	0.445
Current smoker (%)	22.8	16.3	14	0.003**	12.9	14	29.9	0.0001***
Energy intake (kcal/d)	2569±714	2348±707	2401±673	0.068	2564±798	2338±642	2472±654	0.309
Cholesterol (mg/d)^2^	169.84±113.05	200.74±93.13	200.21±78.67	0.0001***	142.85±71.78	189.81±78.56	241.97±102.92	0.0001***
Saturated fat (g/d)^2^	24.01±7.47	23.41±5.35	23.04±6.56	0.523	20.96±5.47	24.12±5.76	25.17±6.02	0.0001***
Monounsaturated fat (g/d)^2^	25.30±7.56	26.32±10.89	24.33±5.35	0.600	23.89±6.54	23.13±5.6	25.70±5.57	0.013*
Polyunsaturated fat (g/d)^2^	15.98±6.44	16.12±5.76	14.01±4.03	0.010*	14.50±5.54	13.31±3.58	15.41±4.80	0.037*
Fiber (g/d)^2^	41.41±20.01	45.17±20.36	48.56±17.40	0.0001***	52.76±16.67	41.32±15.34	37.46±11.51	0.0001***
Protein (g/d)^2^	78.37±14.67	84.49±13.51	95.00±18.40	0.0001***	90.33±16.50	84.74±18.37	84.14±14.97	0.099
Carbohydrate (g/d)^2^	338.78±44.28	339.57±48.08	340.97±35.29	0.903	345.34±39.01	349.15±37.12	329.77±36.86	0.004**

All data were expressed as mean±SD or percentage

One-way ANOVA was used

1 Metabolic equivalent (energy need per kilogram of body weight per hour of activity divided by the energy need per kilogram of body weight per hour at rest) hours per week

2 Energy adjusted

* p<0.05

** p<0.01

*** P<0.001

**Table 4:** Serum concentration of total antioxidant capacity (TAC) and Malondialdehyde (MDA) by quintile of dietary pattern

**Dietary pattern**	**TAC**	**MDA**
Healthy	U/ml	µmol/L
Q1	6.85 (6.37, 7.33)	5.46 (4.13, 6.79)
Q5	9.87 (9.54, 10.24)	3.03 (2.52, 3.54)
P value	0.0001***	0.005**
Unhealthy		
Q1	8.90 (7.66, 10.16)	3.49 (1.96, 5.02)
Q5	6.11 (5.63, 6.60)	6.02 (5.35, 6.69)
P value	0.001**	0.013*

Independent sample t-test was used.

* For the p<0.05,

** p<0.01 and

*** P<0.001.

**Table 5:** β regression coefficients for a one z-score increase in dietary pattern scores

**Biomarkers**	**Healthy**			**Unhealthy**		
	**Model 1**	**Model 2**	**Model 3**	**Model 1**	**Model 2**	**Model 3**
TAC	0.278 (0.0001***)	0.258 (0.005**)	0.244 (0.008**)	-0.378 (0.0001***)	-0.305 (0.0001***)	-0.289 (0.001**)
MDA	-0.213 (0.004**)	-0.274 (0.001**)	-0.273 (0.002**)	0.355 (0.0001***)	0.386 (0.0001***)	0.387 (0.0001***)

*P *values in parentheses.

Model 1 was unadjusted.

Model 2 was adjusted for age, sex, physical activity, and smoking status.

Model 3 was adjusted for all factors in model 2 plus BMI.

Multiple linear regressions analysis was used.

* For the p<0.05,

** p<0.01 and

*** P<0.001.

## Discussion

The main finding of this study was the strong and significant association of TAC with both dietary patterns. TAC levels rose gradually with higher compliance to the healthy diet and a similar negative trend was noted for the unhealthy pattern, indicating that the healthy pattern has higher levels of antioxidants, while the unhealthy diet has lack in antioxidant. These associations remained significant at 2 levels of adjustments for age, sex, physical activity, BMI, and smoking, findings to be expected as followers of the healthy pattern reported more servings of fruits, vegetables and olive products known to be rich in antioxidants and also found in abundance in the Mediterranean diet, which is known to be associated with elevated TAC levels and low ox-LDL levels (18). In a controlled trial, 103 subjects were assigned to consume either typical American diet or a DASH diet, for 3 months, and results concluded that a diet rich in antioxidants (DASH) reduces oxidative stress (19). In a more recent retrospective study in young adults, after observing an inverse association between TAC and glucose and lipid biomarkers as well as adiposity, authors have recommended TAC to be a useful tool for assessing health benefits of cumulative antioxidant capacity from food intake and also have reported an independent and inverse relationship for ox-LDL with plasma TAC (20).

It appears that the relationship between serum MDA and dietary pattern is reported for the first time in this study. Plasma levels of MDA were inversely associated with the healthy pattern, such that with increased compliance with the healthy pattern, MDA levels decreased in a stepwise manner; the compatible relationship was found for plasma MDA and the unhealthy pattern, demonstrating the increase of oxidative stress with greater adherence to unhealthy pattern. Moreover, these associations remained significant after adjustment for potential confounders (age, sex, physical activity, smoking and BMI) which indicates the independent nature of this finding. Data available on relationship between diet and MDA mostly focus on single nutrients or food items. 

A cross-sectional study of HIV-positive patients suggested that among other antioxidants, dietary selenium intake was strongly and inversely associated with plasma MDA (21). In another study, researchers reported that vitamin E supplementation (1.5 mg vitamin E/g body weight of rat) significantly decreased MDA concentrations in acute aluminum phosphide poisoning in rats (22). Another controlled trial of rats showed that MDA excretion increased with higher salt intake (23). In an interventional study design, the effect of antioxidants from fruits and vegetables on antioxidant defense was compared with the equivalent by the supplements, results showed that fruit and vegetables provide more oxidative protection than vitamins and minerals supplements (24). 

The beneficial effects of diet in ameliorating symptoms of MetS have been previously studied (25-27). In a controlled trial addressing the association between a specific dietary pattern and oxidative stress in MetS patients, concluded that the RESMENA (metabolic syndrome reduction in Navarra) diet (30% proteins, 30% lipids, 40% carbohydrates) specifically reduced the android fat mass and demonstrated more effectiveness on improving general oxidative stress; they also noted that ox-LDL values were inversely associated with dietary TAC and fruit consumption (28). Their results suggested that increased amounts of protein and simultaneously lower levels of carbohydrate could be beneficial in decreasing oxidative stress and thus can benefit MetS patients. Their diet was similar to the healthy diet in our study, as in the healthy pattern lower level of carbohydrate consumption was measured, also higher levels of protein intake were reported by the consumers of this pattern. The harmful effects of high carbohydrate intake on MetS have often been reported (25, 29, 30); on the other hand, the potential benefit of higher protein consumption, might be partially explained by the increased levels of uric acid. It is proposed that high protein diets lead to higher levels of uric acid in serum, suggesting that uric acid could have a protective role and could act as an antioxidant in the body (31, 32), highlighting antioxidant role of uric acid in individuals; uric acid is recognized as a powerful antioxidant that scavenges singlet oxygen, oxygen radicals, and peroxynitrite and chelates transition metals. Data show that urate thus accounts for approximately half of the antioxidant capacity of human plasma, and its antioxidant properties are as powerful as those of ascorbic acid (33, 34). 

Nonetheless, the role of uric acid in conditions associated with oxidative stress is not entirely clear and some studies find this pro-oxidant role of uric acid (35). To improve oxidative stress, implementation of specific diet (RESMINA diet; 30% proteins, 30% lipids, 40% carbohydrates) has shown beneficial effects in ameliorating oxidative stress and reducing android fat mass (28). Consumption of a healthy diet deficient in antioxidants is shown to reduce oxidative stress (19); moreover, evaluating the relations between dietary TAC and some MetS features showed an inverse relationship between TAC and a number of MetS symptoms (9). Therefore, a marked affiliation is perceived between dietary pattern, oxidative stress and manifestation of MetS.

A case-control study comparing the oxidative stress levels in patients mostly with type I diabetes with normal controls; reported that in diabetic patients, blood levels of antioxidants are not related to their dietary intakes but to serum lipids; although the low number of subjects (30 subjects) and the fact that most of them had type I diabetes make the results very difficult to generalize, results showed that dietary antioxidants do not have the same effect in all people (36); Therefore, different diseases should be studied separately for the effect of diet on oxidative stress. Several MetS components such as obesity (37), hypertension (38), or type 2 diabetes (39) are characterized by an increase in oxidative stress (40). 

Antioxidants, including vitamins such as b-carotene, vitamin C, and vitamin E, and the minerals selenium and zinc, abundantly found in foods like fruits, vegetables, fish, and olive, can diminish the oxidative process by inactivating free radicals and may provide a protective effect for the organism against MetS and its complications. Nevertheless, some studies show no beneficial effects of antioxidant supplementation on incident of MetS in generally well-nourished populations (38, 41, 42). One hypothesis could be that supplementation alone is not enough to protect the organism and epidemiologic data also confirm that a healthy dietary pattern such as a Mediterranean diet could be more beneficial in preventing MetS and its related components (41). In other words, although dietary patterns seem to be more influential in defining the state of the body with MetS, more well-designed studies are required to confirm current data on this topic. 

This study has some limitations. One of which is the use of FFQ that relies on the long term memory of individuals, and exposes the outcome to memory bias. Although this FFQ has previously been validated in the same population, applying a predefined FFQ limits the number of food items for subjects. Our budget limitation forced us to limit the number of enrolled subjects in this study; however, the longitudinal design of this study is a major strength. This design removes many of the biases seen in other designs, such as cross-sectional, because subjects are unaware of any change in their oxidative stress status when they report dietary intake. 

In conclusion, according to our results the healthy dietary pattern with plenty of fruits and vegetables can ameliorate oxidative stress status by reducing MDA and increasing TAC and on the contrary, adherence to the unhealthy pattern, characterized by the higher intakes of fast foods and processed foods, aggravated the oxidative stress levels in Tehranian individuals suffering from metabolic syndrome. 
